# Effects of Process Parameters on the Characteristics of Mixed-Halide Perovskite Solar Cells Fabricated by One-Step and Two-Step Sequential Coating

**DOI:** 10.1186/s11671-016-1601-8

**Published:** 2016-09-17

**Authors:** Mohammad Reza Ahmadian-Yazdi, Fatemeh Zabihi, Mehran Habibi, Morteza Eslamian

**Affiliations:** University of Michigan-Shanghai Jiao Tong University Joint Institute, Shanghai, 200240 China

**Keywords:** Photovoltaics, Mixed-halide perovskite, Sequential deposition, Crystallization dewetting, One-step deposition

## Abstract

In this paper, two-step sequential spin-dip and spin-spin coating, as well as one-step spin coating, methods are used to fabricate methylammonium lead mixed-halide perovskites to study the effect of process parameters, including the choice of the solvent, annealing temperature, spin velocity, and dipping time on the characteristics of the perovskite film. Our results show that using a mixture of DMF and DMSO, with volume ratio of 1:1, as the organic solvents for PbCl_2_ results in the best mixed-halide perovskite because of the effective coordination between DMSO and PbCl_2_. Surface dewetting due to two effects, i.e., crystallization and thin liquid film instability, is observed and discussed, where an intermediate spin velocity of about 4000 rpm is found suitable to suppress dewetting. The perovskite film fabricated using the one-step method followed by anti-solvent treatment shows the best perovskite conversion in XRD patterns, and the planar device fabricated using the same method exhibited the highest efficiency among the employed methods. The perovskite layer made by sequential spin-dip coating is found thicker with higher absorbance, but the device shows a lower efficiency because of the challenges associated with perovskite conversion in the sequential method. The one-step deposition method is found easier to control and more promising than the sequential deposition methods.

## Background

Lead halide perovskite solar cells have drawn enormous attention due to their rapid rise in device power conversion efficiency (PCE), only within a few years, now hitting a record PCE of 22 % achieved by Seok’s group (KRICT/UNIST, South Korea), although the device was not stabilized [1]. The perovskite crystal structure has the chemical formula of ABX_3_, where in the perovskite molecule used in solar cells as the light-harvesting layer, A is usually methylammonium, B is lead, and X is a halogen or a mixture of several halogens, thus making the CH_3_NH_3_PbX_3_ molecule with proper light absorbance and bandgap suitable for photovoltaic applications [2, 3]. Miyasaka was the first who employed it as a sensitizer layer in dye-sensitized solar cells and achieved a PCE of 3.8 % [4]. Since then, attempts have been made to engineer the device structure to increase the efficiency and stability [5]. Methylammonium lead halide perovskite usually forms according to the following chemical reaction between its two precursors:

1 $$ {\mathrm{PbX}}_2+{\mathrm{CH}}_3{\mathrm{NH}}_3\mathrm{X}\overset{\mathrm{Organic}\ \mathrm{solvents},\ \mathrm{Temp}.}{\to }{\mathrm{CH}}_3{\mathrm{NH}}_3{\mathrm{PbX}}_3 $$

where X is a halogen, usually I or Cl, which could be the same in both precursors or different. Both the single-halide CH_3_NH_3_PbI_3_ and mixed-halide CH_3_NH_3_PbI_3 − *x*_Cl_*x*_ perovskite molecules have been widely used as the light-harvesting layer in perovskite solar cells (SCs) [6]. However, it has been demonstrated that the mixed-halide perovskite exhibits superior photovoltaic performance, such as small exciton binding energy [7], high charge-carrier mobility, and large exciton diffusion length over 1 μm, which makes it compatible with various cell structures. Compared to CH_3_NH_3_PbI_3_, the chlorine ion in mixed-halide perovskite CH_3_NH_3_PbI_3 − *x*_Cl_*x*_ improves the charge-carrier transport [8, 9], the charge collection of the cell, and the band bending, and lowers the charge recombination rate. It is, however, difficult to tune the chemistry of the chlorine-doped perovskite, as it requires solvent engineering and well-controlled deposition methods. This entails more research on mixed-halide perovskites to understand the perovskite conversion process and the film characteristics.

Given that perovskite has a crystalline structure, thin layers of perovskite used in solar cells are prone to dewetting because of the growth of rather large crystals on the film, adversely affecting the film intactness and coverage. Significant progress has been made in the film morphology and surface coverage; however, the perovskite films still suffer from rough and non-uniform morphologies [10], indicating the need for more studies to understand the process and to obtain pinhole-free films.

As Eq. (1) suggests, the perovskite can be fabricated using a wet chemistry process. The first solution-processed perovskite layer (CH_3_NH_3_PbI) was synthesized on a mesoporous TiO_2_ scaffold and incorporated into an electron/hole-selective-free device [4]. The mesoporous scaffold made of nanoparticles of TiO_2_ helps in the controlled growth of perovskite crystals. The mesoporous scaffold filled with perovskite crystals also helps in the effective charge collection in a short distance, which is within the diffusion length of the charges. This structure was then developed by addition of electron- and hole-transporting layers and replacing the TiO_2_ scaffold by other ceramics, such as Al_2_O_3_. These mesoporous 3D structures are known as the typical structure for perovskite SCs, but they essentially need high process temperatures for their fabrication, hindering low-cost fabrication of perovskite SCs, especially on flexible plastic substrates. To suppress the difficulties associated with mesoporous structures, an alternative structure has been suggested, based on deposition of perovskite reagents on flat substrates, called planar or 2D structure [11]. The planar structure is better matched with the properties of the mixed-halide perovskites, which possess longer charge diffusion lengths compared to single-halide perovskites. Planar perovskite devices can be fully processed in solution and deposited using a casting method. However, in a planar structure, the elimination of the mesoporous scaffold, which functions as a template for crystal growth, complicates the controlled deposition, chemical conversion, crystal growth orientation, and may cause hysteresis at the interfaces. To suppress these difficulties, several attempts have been made to control the crystal growth and improve the interfaces of perovskite with the adjacent layers by employing various deposition methods, such as vapor deposition or solution-processed casting methods, e.g., [11–19]. The deposition method affects the characteristics of the resulting thin films, i.e., the coverage, thickness, roughness, and therefore the light-harvesting and charge-carrier pathways. Perovskite layers may be deposited sequentially in two steps, given that two precursors are involved in the fabrication of perovskite (Eq. 1), or in one step, by mixing and depositing the precursors. Each approach has its advantages and disadvantages. For instance, although some works indicate the supremacy of the sequential deposition, other studies reveal that the perovskite thin film deposited on a flat substrate may result in incomplete conversion of precursors to perovskite [20], due to inadequate mixing of precursors in the sequential deposition.

Regardless of the fabrication route, understating and controlling the process parameters, such as annealing temperature, concentration of precursors, and employed solvents and additives, play critical roles in the film surface quality, coverage, conversion of precursors to perovskite, crystallinity, crystal size, and therefore the performance of the layer and the entire device [21]. Following the forgoing introduction, the main goal of this work is to understand the effect of the aforementioned parameters on perovskite film and device performance. The planar structure is adopted for its low-temperature processing, and the mixed-halide perovskite is chosen for its better performance, longer charge diffusion length, and compatibility with planar structure. By varying several process parameters, we attempt to obtain the optimum conditions. Three fabrication routes are followed to make perovskite layers: two-step sequential spin-dip and spin-spin coating and one-step spin coating. The solar cells, made based on the optimum condition, have a planar FTO-coated glass/c-TiO_2_/perovskite/spiro-OMeTAD/Ag structure, where FTO denotes fluorine-doped tin oxide, c-TiO_2_ denotes compact TiO_2_ film, and spiro-OMeTAD is the short form for 2,2′,7,7′-tetrakis(*N*,*N*′-di-*p*-methoxyphenylamine)-9,9′-spirobifluorene.

## Methods

Methylammonium iodide (MAI, 99.5 %) was supplied by Xia’an Reagents Co., China. 2-Propanol (IPA, anhydrous, 99.5 %), *N*,*N*-dimethyl formamide (DMF, 99.8 %, biological grade), chlorobenzene (CB, 99.9 %, analytical grade), dimethyl sulfoxide (DMSO, 99.5 %, biological grade), hydrogen chloride (HCl, 37.5 %), lead chloride (PbCl_2_, 98.5 %), ethanol (99.9 %, biological grade), toluene (98.8 %), bis(trifluoromethane)sulfonamide lithium salt (99.95 %), acetonitrile (99.8 %), 4-tert-butylpyridine (96 %), and titanium (IV) isopropoxide (97 %) were purchased from Sigma-Aldrich, USA.

Fluorine-doped tin oxide (FTO)-coated glass (25 mm × 25 mm × 2.2 mm) was washed by detergent, deionized water, and 2-propanol in an ultrasonic bath for 30 min, followed by UV-ozone treatment for 15 min. For the preparation of compact TiO_2_ layer (c-TiO_2_), as the electron-transporting layer (ETL), 350 μl of titanium isopropoxide solution was diluted in 5 ml of ethanol. Also, 32.5 μl of HCl (2 M) was diluted in 2 ml of ethanol. Then, the acidic solution was added to titanium isopropoxide solution dropwise, under stirring condition. The resultant solution was then cast by two methods: spray coating and spin coating at 2000 rpm for 60 s. The resulting thin films were annealed at 150 °C, for 30 min. The spray-on c-TiO_2_ thin films were rough and demonstrated low device performance, when used for device fabrication, and therefore were discarded.

Perovskite thin films were prepared using one-step and also two-step sequential casting of precursor solutions: PbCl_2_ was dissolved in DMF, DMSO, and a mixture of both solvents at various concentrations (c.f. Table 1), while MAI was dissolved in sufficient amount of IPA to obtain a concentration of 10 mg/ml. In order to investigate the influence of the choice of solvent on thin film quality and surface coverage and morphology of crystals, PbCl_2_ was dissolved in various ratios of DMSO to DMF solvents. In the spin-dip two-step sequential coating, the PbCl_2_ solution was spun for 60 s, at various spin speeds of 1000, 4000, and 7000 rpm. The resulting wet film was then heat-treated at 90 °C for several seconds and was immediately dipped into the MAI solution and kept for 90 or 150 s to study the effect of dipping time. As-spun thin films were annealed at various temperatures of 100 and 160 °C, for 30 min. In the spin-spin two-step sequential coating, the PbCl_2_ film was made by the same method just described for the spin-dip process, and then, the MAI solution was spun over at 2000 rpm for 40 s. Then, the wet film was heat-treated at 90 °C for 1 h. Due to the hygroscopic nature of MAI and instability of the CH_3_NH_3_PbI_3 − *x*_Cl_*x*_ film in ambient conditions, all samples were fabricated in a nitrogen-filled glove box.

**Table 1:** The list of experimental runs and variables used in this study. The measured roughness values of perovskite films are also listed

Run #	Concentration of PbCl_2_ (mg/ml)	Annealing temp. (°C)	Spin velocity (rpm)		Dipping time (s)	DMSO vol. (ml)	DMF vol. (ml)	Roughness (μm)	Coating method
1^†^	200	100	4000		90	2	2	0.10	Spin-dip
2	200	160	4000		90	2	2	0.20	Spin-dip
3	200	100	7000		150	2	2	0.20	Spin-dip
4	200	100	1000		150	2	2	0.20	Spin-dip
5	200	100	4000		150	2	2	0.25	Spin-dip
6	267	100	4000		90	2	1	0.26	Spin-dip
7	267	100	1000		90	1	0	0.10	Spin-dip
8	150	100	4000		150	2	2	0.13	Spin-dip
9	150	100	4000		90	2	2	0.10	Spin-dip
10	200	100	4000^a^	2000^b^	–	2	2	0.67	Spin-spin
11^c^	–	100	–		–	0	1	0.20	One-step

^†^Run 1 was found to be the optimum case. For XRD device fabrication and XRD analysis, after deposition of PbCl_2_, a layer of PbI_2_ was also deposited to further improve the perovskite coverage and conversion, as outlined in the “Methods” section

^a^Spin velocity for deposition of PbCl_2_ precursor

^b^Spin velocity for deposition of MAI precursor

^c^Details are given in the “Methods” section

For the one-step deposition, the perovskite precursor solution was prepared by dissolving 481 mg of PbI_2_, 40 mg of PbCl_2_, and 100 mg of MAI in 1 ml of DMSO. The solution was spun for 20 s at 750 rpm and then immediately for 60 s at 3000 rpm. After 40 s, 300 μl of toluene was dropped onto the thin film and the obtained wet film was annealed at 100 °C for 10 min.

Devices were fabricated using the perovskite films made based on the two-step sequential spin-dip method at optimum process conditions (run 1 of Table 1), as well as the one-step method. To deposit the hole-transporting layer (HTL), 60 mg of spiro-OMeTAD, 480 mg/ml of lithium salt in acetonitrile, and 20 μl of 4-tert-butylpyridine were added to 1 ml of chlorobenzene and the solution was spun at 2000 rpm for 30 s. The wet film was kept for 12 h under nitrogen atmosphere. To complete the device, 200 nm of Ag was deposited by thermal evaporation. The active area of the cells made in this work is about 0.076 cm^2^. To further improve the perovskite conversion of the optimum run 1 of spin-dip coating for X-ray diffraction (XRD) analysis and device fabrication, after spinning of PbCl_2_, as mentioned above, a PbI_2_ solution (10 mg/ml in DMSO) was spun over at 5000 for 30 s.

Surface topography of the perovskite thin films were characterized using a confocal laser scanning microscope (CLSM, model LMS700, Zeiss, Germany) and a scanning electron microscope (SEM, Hitachi, Model S-3400N, Japan). The surface roughness was obtained using the CLSM over an area of 65 μm × 65 μm. A UV-vis spectrometer (Lambda 20, PerkinElmer Inc., USA) was used to obtain the absorbance spectra. XRD (model D5005, Bruker, Germany) was utilized to characterize the crystallographic patterns of thin films. Average perovskite particle (crystal) size was measured by Zetasizer (Malvern, nano-zs90). Interaction of DMSO and DMF solvents with PbCl_2_ was analyzed using X-ray photoelectron spectrometry (XPS, Kratos, Axis ultra DLD). The device photovoltaic performance and the *J*-*V* curves were obtained using a solar simulator and a Keithley source meter, model 2450, the Netherlands, under AM1.5G solar irradiation at a power intensity of 100 mW/cm^2^, in the range of 0 to +1 V bias.

## Results and Discussion

Table 1 lists the process parameters, i.e., the annealing temperature, concentration of PbCl_2_, type and ratio of the organic solvents used to dissolve PbCl_2_, spin velocity, and the perovskite deposition method, as well as the roughness of the perovskite films, fabricated in a nitrogen-filled glove box. Most samples listed in Table 1 were made by the two-step sequential spin-dip coating, one sample listed was made by the two-step sequential spin-spin coating, and one sample was made by the one-step deposition. In the following sub-sections, using the data listed in Table 1 and proper figures, the effects of various process parameters on some characteristics of perovskite films are investigated.

**Fig. 1:**
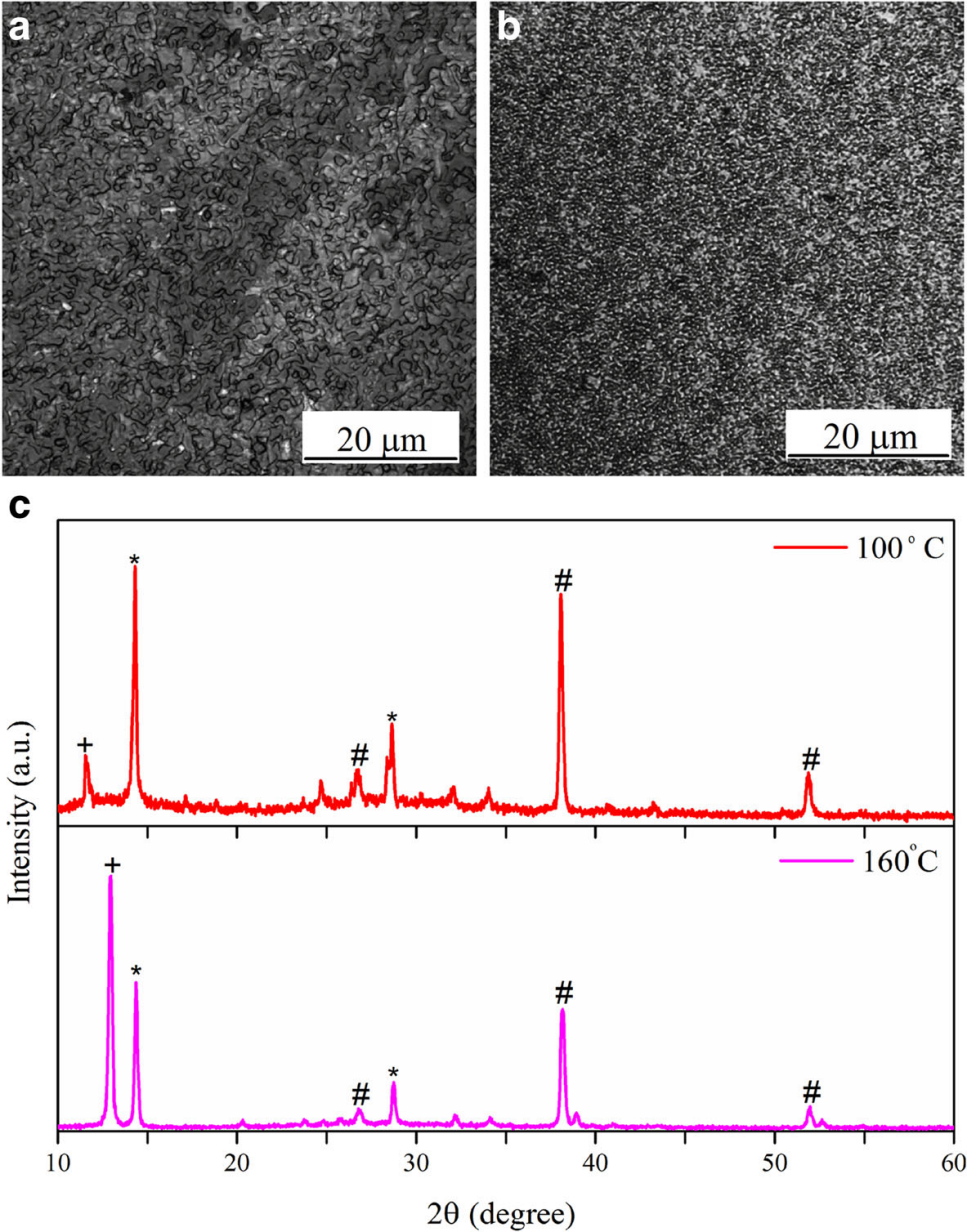
Effect of annealing temperature: laser images of perovskite surface morphology, made using spin-dip coating at **a** annealing temperature of 100 °C (run 1) and **b** annealing temperature of 160 °C (run 2). The field of view is 65 μm × 65 μm. **c** The XRD patterns of perovskites annealed at 100 and 160 °C. The *asterisk* denotes the perovskite peaks, the *number sign* denotes the FTO peaks, and the *plus sign* denotes the PbI_2_ peaks. Annealing at 100 °C is preferred because of better perovskite conversion and adequate coverage

Annealing is an important step performed after deposition of perovskite films, to tune the film crystallinity, to eliminate solvent residues, and to stabilize the perovskite structure. Here, we studied how temperature could affect the perovskite conversion and also distribution and uniformity of perovskite crystals. The laser images of perovskite films made by spin-dip coating using annealing temperatures of 100 and 160 °C (runs 1 and 2) are illustrated in Fig. 1a, b. Two different morphologies are observed. At higher temperature of 160 °C, perovskite crystals are apparently more uniformly distributed. Upon casting of the precursor solutions, an organic/halide-rich wet film forms, where the annealing process helps the excess organic and halide components evaporate, until a stoichiometric ratio of organic:metal:halide is reached [22]. At a higher annealing temperature, the evaporation rate is faster resulting in smaller and more uniform crystals because high evaporation rate arrests excessive crystal growth. However, it was observed that at annealing temperatures higher than 170 °C, the surface coverage decreases, due to aggregation or sintering of perovskite crystals in severe thermal conditions (data not shown). It was also inferred that annealing at very high temperatures results in degradation of perovskite, evidenced by appearing shiny yellow color sites on the surface, indicating the formation of PbI_2_ crystals. In agreement with this argument, Fig. 1c shows that annealing at 160 °C, compared to 100 °C, results in partial decomposition of perovskite. Therefore, overall, it is concluded that annealing at 100 °C is preferred and therefore used here for device fabrication.

**Fig. 2:**
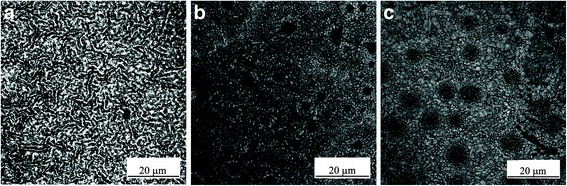
Effect of spin velocity: laser images of spin-dip perovskites. The PbCl_2_ films spun at **a** 1000 rpm (run 4), **b** 4000 rpm (run 5), and **c** 7000 rpm (run 3). All other process parameters were kept constant. The field of view is 65 μm × 65 μm

The laser images of the films made at the conditions of runs 3, 4, and 5 to study the effect of spin velocity and therefore the film thickness on the overall film morphology are shown in Fig. 2, where surface features including pinholes can be observed. As the spin velocity increases, the liquid film thickness decreases, and if the liquid film becomes very thin (less than 100 nm or so), the intermolecular forces, such as van der Waals forces, between the liquid film interfaces (liquid-air and solid-liquid) become dominant and larger than the stabilizing gravity and capillary forces due to surface tension, and the film may become prone to spinodal dewetting, given that ultrathin films are usually metastable. In addition, a thin liquid film may partially break up and dewet the surface, due to heterogeneous nucleation, induced by the presence of impurities on the substrate or within the film. Such heterogeneous nucleation may be induced by thermal effects, as well [23]. Besides the conventional dewetting of thin liquid films that may occur due to the growth of perturbations, dewetting in evaporating thin films of liquid solutions may also occur due to crystallization dewetting, i.e., the formation of large crystals that consume the solid content, thus creating dewetted areas adjacent to the large crystals [24]. A thin liquid film of perovskite precursor solution is a perfect example that shows the occurrence of crystallization dewetting, due to the growth of large crystals of perovskite. In summary, at high spin speeds or low film thickness, the chance of dewetting due to crystallization as well as breakup due to excessive growth of liquid film perturbations increases, as observed clearly in Fig. 2c (7000 rpm) and partially in Fig. 2b (4000 rpm). In Fig. 2, the PbCl_2_ solution was spun for 60 s. The relationship between the estimated film thickness and spin velocity and duration of spinning may be found in Ref. [23].

**Fig. 3:**
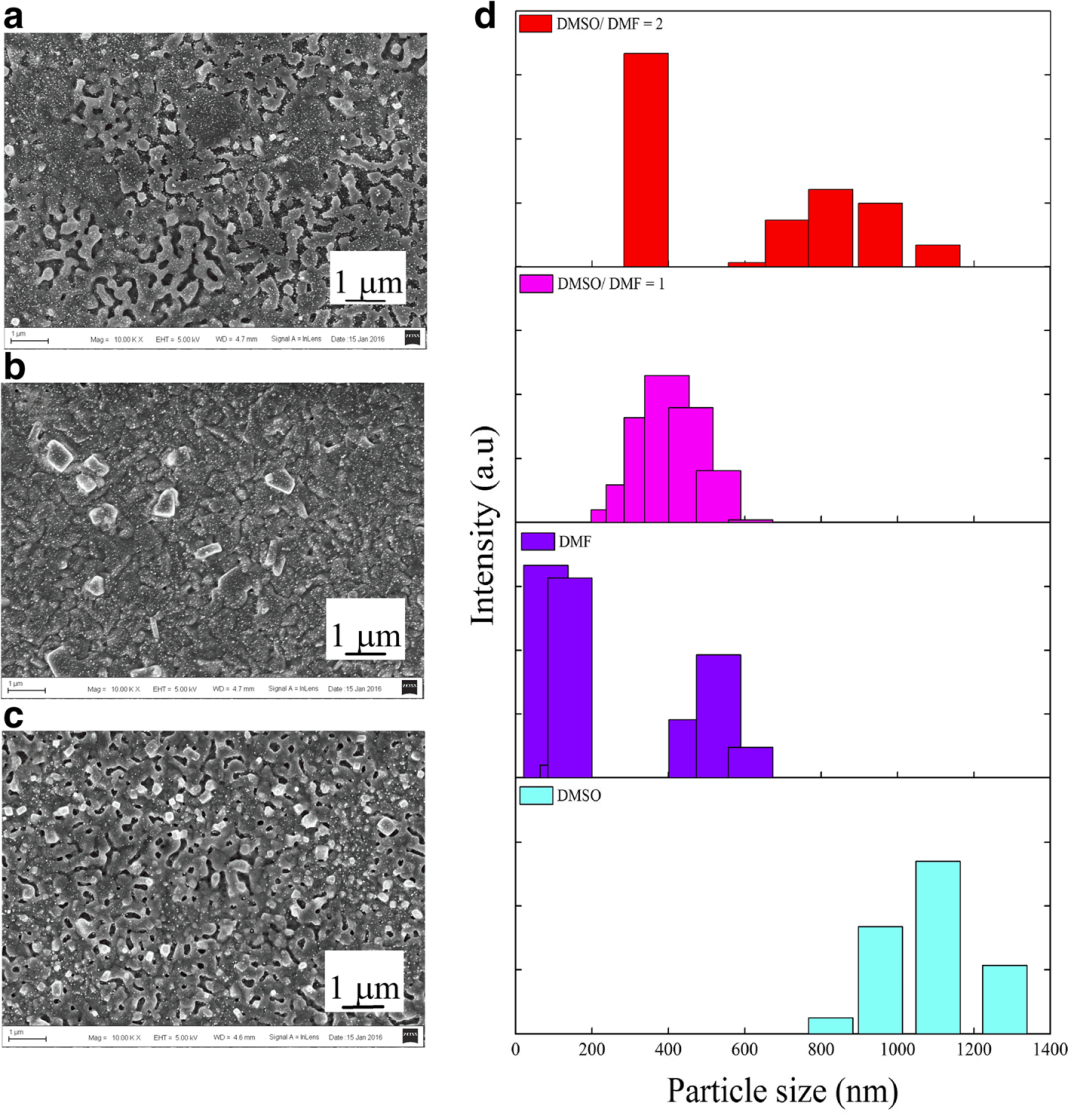
Effect of the PbCl_2_ solvent: SEM images of perovskite films made by spin-dip coating method, using various solvents for PbCl_2_: **a** DMSO/DMF = 2 (run 6), **b** DMSO/DMF = 1 (run 1), **c** DMSO only (run 7), and **d** perovskite particle size distribution for various PbCl_2_ solvents

To promote perovskite conversion and to control the kinetics of crystal growth and to avoid dewetting, various approaches are employed, including solvent treatment. A strong coordination between the solvent and the solute is the determining factor for selection of a favorable solvent. A good coordination between the solvent and solute is beneficial for effective distribution of the solute in the solvent and therefore uniform crystallization and coverage of the film [25]. Also, the choice of the solvent usually affects the crystal size [24]. Most studies indicate that larger crystal size (e.g., those obtained by DMSO-based solution) favors the light absorbance, e.g., [26]. However, the large crystals tend to cause crystallization dewetting and adversely affect the film coverage, which causes frequent shunt paths between perovskite and the adjacent layers. Here, the effect of using DMF and DMSO solvents on the coverage of perovskite thin films is investigated (Fig. 3). We found that DMF is a rather poor solvent for PbCl_2_, whereas DMSO better dissolves PbCl_2_. On the other hand, it has been shown that with DMF, perovskite formation occurs immediately after solvent evaporation, within a few minutes of annealing, while DMSO retards the reaction, because of the formation of strong DMSO-Pb-Cl- complexes [10, 13]. We speculated that using a mixture of DMF and DMSO would result in a controlled formation of perovskite and better coverage. Figure 3 shows the SEM images and crystal size distribution of perovskite layers made using DMSO and a mixture of DMSO and DMF solvents. The average perovskite crystal size and the size distribution were measured by a Malvern Zetasizer. Particle sizing is sometimes performed by analysis of SEM or optical images, but the particle sizer is more reliable and accurate. In image analysis, the image size and resolution may be limited; it may be also difficult to recognize every individual grains, due to possible crystal agglomeration or overlapping in some samples. For particle sizing by Malvern Zetasizer, the whole sample is dispersed in a dispersant. Then, the crystals are separated or agglomerates are broken by agitation or sonication. Therefore, a large number of particles are subjected to particle counting. Figure 3d shows that the application of DMSO results in the formation of larger crystals compared to those made using DMF. As observed from the SEM images, the highest coverage is obtained when a mixture of DMSO and DMF with a volume ratio of 1 is employed (run 1). Also, the particle size distribution graphs (Fig. 3d) reveal that the application of the same mixture results in the formation of smaller crystals with a narrow size distribution compared with the case of using DMSO only. Application of a mixture of DMF and DMSO with a volume ratio of DMSO/DMF = 2 results in a bimodal size distribution with lower coverage. From a hydrodynamics point of view, addition of DMF to DMSO increases the solution surface tension, thus improving the thin liquid film stability and uniform coverage of the resulting solid film. Besides, addition of DMF decreases the polarity of the solution. In the case of partial drying, a residual polar solvent may boost the hydration of perovskite crystals.

**Fig. 4:**
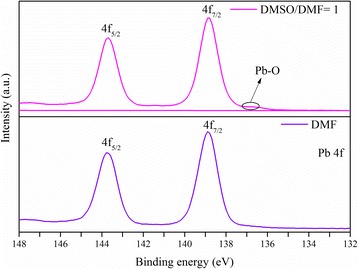
XPS of PbCl_2_ dissolved in different solvents

Literature reports show that strong coordinating solvents, such as DMSO, facilitate the formation of a uniform film of PbI_2_ crystals [27, 28]; however, there is no report on the same effect on PbCl_2_, and thus, it is studied here by XPS analysis, as shown in Fig. 4. In Fig. 4, the located high peaks at 138.7 and 143.5 eV are associated with PbCl_2_ bonds [27]. As shown in Fig. 4, the PbCl_2_ sample made using DMF has only two peaks associated with PbCl_2_, whereas the sample made using a mixture of DMF and DMSO has an additional low-intensity Pb-O peak, since the polarity of DMSO is larger than that of DMF. Furthermore, because of the high polarity of DMSO, which is the result of the bond between S and O, DMSO can make covalent bonding with PbCl_2_. This behavior of PbCl_2_ in mixed DMSO/DMF solvent is similar to that of PbI_2_, observed before by others, i.e. the PbI_2_ is more uniform and homogenous in the solution, in the presence of both DMSO and DMF [27, 28]. With an increase in the volume of DMSO, the coordination of PbCl_2_ with DMSO increases, which favors the formation of PbCl_2_ clusters. Therefore, the crystals further grow, but excessive amount of DMSO may cause the formation of needle-shape crystals and a decrease in the coverage of the perovskite layer, as observed in the SEM images of Fig. 3.

**Fig. 5:**
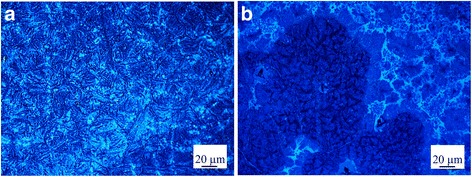
Optical images on the effect of dipping time on the coverage and morphology of spin-dip perovskites: **a** 90 s (run 9) and **b** 150 s (run 8)

Based on our preliminary experiments and the literature data, two reasonable dipping times of 90 and 150 s were selected in spin-dip experiments, to study its effect on the surface coverage and roughness. Increasing the dipping time of the PbCl_2_ layer in the MAI solution, generally, provides longer time for conversion of precursors to perovskite crystals. However, excessive exposure times may cause dissolution of the perovskite layer, leading to film deterioration or a decrease in coverage. Figure 5 shows the effect of dipping time on samples made at 4000 rpm and PbCl_2_ concentration of 150 mg/ml for two different experimental conditions (runs 8 and 9). One observation made here is that the spin-dip perovskite made based on 90 s dipping time is more uniform and appears single-phase, whereas the sample made based on 150 s appears two-phase and affected by the excessive dipping time. Comparing run 8 (*t* = 150 s) with run 9 (*t* = 90 s) of Table 1 reveals that a longer dipping time results in a higher roughness. A higher roughness may translate to a larger crystal size. It is therefore concluded that within the range of the parameters studied here, the lower dipping time (90 s) is preferred. Furthermore, it has been inferred that in the formation of the mixed-halide perovskites, such as the current work, CH_3_NH_3_ICl forms as an intermediate component, which may convert to CH_3_NH_3_PbI_3_ in a long contact time, as exhibited by the following chemical reactions [29]:

1. PbI_2_Cl_2_ + 3CH_3_NH_3_I → PbI_2_ + 2CH_3_NH_3_Cl + 2CH_3_NH_3_I → 2CH_3_NH_3_PbI_3_ + 2CH_3_NH_3_Cl(g)↑
2. PbI_2_ + *x*CH_3_NH_3_I + *y*CH_3_NH_3_Cl → (CH_3_NH_3_)_*x* − *y*_ PbI_2_Cl_*y*_ (Intermediate) → CH_3_NH_3_PbI_3_ + CH_3_NH_3_Cl (g)↑

A long contact time between the spun PbCl_2_ film and the MAI solution may lead to the formation of CH_3_NH_3_PbI_3_, and therefore the release of the CH_3_NH_3_Cl gas. Therefore, the new phase observed in Fig. 5b may be due to the prolonged contact between the precursors, which might have led to the formation of CH_3_NH_3_PbI_3_.

Spin-dip process usually leads to the formation of thicker perovskite films. Although the thicker layer of perovskite may lead to improved light absorption, the film thickness cannot exceed the charge diffusion length. Therefore, alternatively, spin-spin coating may be adopted, instead of spin-dip coating, to make thinner layers. In this method, the dipping process is simply replaced by a second spinning process to deposit a thin layer of MAI solution atop the PbCl_2_ layer. Dynamic interaction and mixing between solid PbCl_2_ and wet MAI layers, due to spinning, results in the formation of perovskite. Run 10 of Table 1 shows the conditions of the spin-spin sample, determined based on the literature data and our preliminary optimization process. In order to enable high solvent evaporation, the substrate was heated before precursor deposition. Therefore, a stable perovskite layer formed, but the film had a large roughness of 0.67 μm, higher than those observed in spin-dip samples (Table 1). It is also found that, in general, at similar process and fabrication conditions, the surface topography of the film made by spin-spin coating is similar to the topography of the film made by spin-dip coating (figure not shown). It was also observed that in spin-spin coating, when the MAI solution was spun atop the spun-on PbCl_2_ thin film, the color changed to dark brown even before annealing, indicating that the perovskite layer had formed. Therefore, perovskite conversion in spin-spin coating is achieved easier than that in spin-dip coating, due to the positive effect of the centrifugal forces that improve mixing of the precursors and therefore lead to better chemical conversion. On the other hand, the presence of the centrifugal forces applied on the wet perovskite film may promote dewetting; nevertheless, by controlling the spin velocity, the dewetted areas and defects may be decreased.

The previous section reveals the difficulties associated with controlling the two-step sequential deposition of perovskite, simply because of the involvement of numerous process parameters. Therefore, in this study, the one-step spin coating was also applied to fabricate perovskite films (run 11). The mixture of perovskite precursors was spun at two spin speeds: first at a low speed (750 rpm for 20 s) to allow nucleation of perovskite crystals, immediately followed by spinning at a higher speed (3000 rpm for 60 s) to complete the film formation process. This one-step method is straightforward and preferred, if desired coverage and conversion can be obtained.

**Fig. 6:**
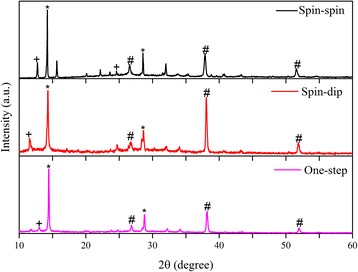
The XRD patterns of perovskites, deposited by spin-dip (run 1), spin-spin (run 10), and one-step (run 11) methods. The *asterisk* denotes the perovskite peaks, the *number sign* denotes the FTO peaks, and the *plus sign* denotes the PbI_2_ peaks

**Fig. 7:**
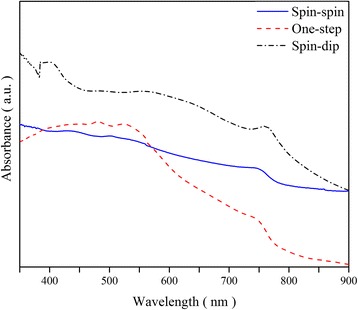
Absorbance of three perovskite films: two-step sequential spin-dip coating (run 1), two-step sequential spin-spin coating (run 10), and one-step spin coating deposition (run 11)

To compare the conversion of precursors to perovskite, the XRD patterns of the best sequential spin-dip (run 1), spin-spin (run 10), and one-step (run 11) spin-coated perovskites are shown in Fig. 6. The background FTO peaks are due to the FTO-coated glass substrate and may be disregarded. The perovskite peaks are observed in all samples. Also, there is no layer of MAI left on the film surfaces as the peaks suggest, indicating effective conversion of MAI to perovskite. The XRD pattern of the sample made by spin-spin coating reveals that the conversion of the precursor solutions to perovskite crystals has increased compared to the spin-dip film of run 1. The film made using the one-step deposition shows the best perovskite conversion, although PbI_2_ peaks are not completely removed. Figure 7 shows the absorbance of the perovskite layers produced by sequential spin-dip and spin-spin coating, as well as one-step spin coating. All films show a drop in the absorbance around the wavelength of 750 nm, which is the characteristic of perovskite layers due to their bandgap, because at large wavelengths, the low-energy photons cannot be absorbed by perovskite. The needle-like structure of the mixed-halide perovskite has been argued to be responsible for good absorbance at low wavelengths, due to internal light scattering of this kind of structure [30]. Although poor coverage of perovskite layer could lead to a decrease in absorbance, this specific structure and the light scattering in the crystals enhances the absorbance. The spin-dip sample prepared based on the conditions of run 1 exhibits the best absorbance in a wide range of wavelengths. The sample made using the one-step spin coating based on the conditions of run 11 shows good absorbance at low wavelengths, but its absorbance declines at wavelengths higher than 550 nm. It is deduced that the spin-dip perovskite is thicker than the one-step spin-coated perovskite, resulting in better absorbance in the spin-dip sample. The dipping process results in the formation of thicker films. In summary, the XRD patterns (c.f. Fig. 6) show the best conversion for the one-step deposition, whereas the absorbance data of Fig. 7 show that the spin-dip sample has a higher absorbance.

**Fig. 8:**
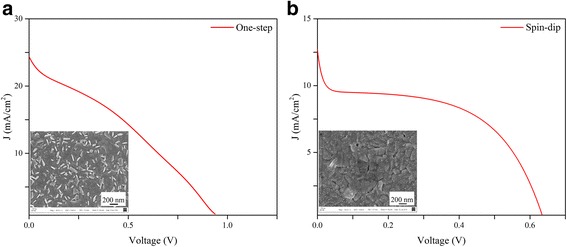
*J*-*V* curves of perovskite solar cells made using **a** one-step spin coating (run 11) and **b** two-step sequential spin-dip deposition based on run 1

Based on the conclusions of the forgoing fundamental study on the characteristics of perovskites made using one-step and two-step sequential methods, it is inferred that the spin-dip perovskite film made based on run 1 and the perovskite film made based on the one-step spin coating method have the best overall coverage, conversion, and absorbance. Thus, these perovskite films were incorporated into planar perovskite SCs. Figure 8 displays the *J*-*V* curves and SEM images at the inset showing the coverage, and Table 2 lists the summary of the performance of the fabricated cells. Although the perovskite and other layers were made in a controlled environment in nitrogen-filled glove box, for deposition of electrodes by thermal evaporation, and also for performance tests, the cells were exposed to humid air of Shanghai (relative humidity in Shanghai is higher than 50 % most of the time). It is well known that high humidity is detrimental to the stability and performance of perovskite SCs. Bearing these difficulties in mind, a moderate PCE of 7.26 % for the one-step cell and a PCE of 3.47 % for the spin-dip cell was recorded. The *J*-*V* curve of the spin-dip device shows anomalous behavior close to the short circuit condition, where the slope of the curve and therefore the shunt resistance changes significantly. Overall, the devices fabricated using the two-step sequential spin-dip coating of run 1 were found rather non-reproducible due to the lack of adequate control on the dipping process. The higher PCE of the one-step cell is attributed to better perovskite conversion, as evidenced from the XRD patterns (c.f. Fig. 6), and better charge collection, even though the absorbance of the one-step perovskite was lower than that of the spin-dip perovskite (c.f. Fig. 7).

**Table 2:** Photovoltaic performance of devices made by two-step sequential spin-dip (run 1) and one-step deposition

	*J*_sc_ (mA cm^−2^)	*V*_oc_ (V)	Fill factor	PCE (%)
Spin-dip (run 1)	12.8	0.63	0.43	3.47
One-step (run 11)	24.38	0.93	0.32	7.26

**Fig. 9:**
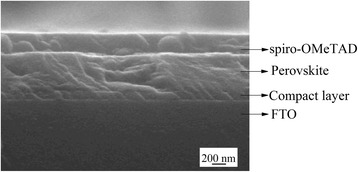
SEM image of the perovskite SC, fabricated by one-step spin coating (run 11). The relative thickness of each layer can be deduced from the scale bar

Figure 9 shows the SEM cross-sectional image of the cell fabricated using the one-step spin coating method. The thickness of the c-TiO_2_, perovskite, and spiro-OMeTAD layers are approximately 70, 350, and 200 nm, respectively. The SEM image shows uniform film thickness and good interfaces between adjacent layers. The perovskite thickness of the one-step device is in the ideal range as corroborated by Docampo et al. [31], who obtained the best device performance for perovskite thicknesses in the range of 300–400 nm, enabling the active layer to absorb near 90 % of the incident photons between 350 and 750 nm. Increasing the film thickness to 600 nm exhibited 99 % light absorbance but caused a drop in the short circuit current. A drop in the short circuit current of the spin-dip device compared to that of the one-step spin-coated device (c.f. Fig. 8) is therefore attributed to the increased thickness of the perovskite layer made by the spin-dip process and/or inadequate perovskite conversion. In summary, we found that the one-step fabrication method is preferred, due to the ease of fabrication, better control on the process, fewer parameters to control, and higher device performance and reproducibility.

## Conclusions

In this work, the characteristics of two-step sequential spin-dip- and spin-spin-coated, as well as one-step spin-coated, mixed-halide perovskite films were studied. In particular, in the spin-dip method, the effect of annealing temperature, choice of solvent for PbCl_2_, dipping time, and spin velocity on the surface topography and roughness, conversion of precursor solutions to perovskite crystals, and light absorbance was studied. Planar devices based on the FTO-coated glass/c-TiO_2_/perovskite/spiro-OMeTAD/Ag structure were fabricated using two-step spin-dip and one-step spin coating methods.

It was found that higher annealing temperature (160 °C) produces a more uniform perovskite film compared to a lower temperature (100 °C). However, high temperature may result in sintering of perovskite crystals. Therefore, within the range of the temperatures studied here, an annealing temperature of 100 °C was found to be the optimum annealing temperature for the best overall performance. Two types of dewetting in perovskite films were observed and discussed in this work. These include dewetting due to the growth of perovskite crystals (crystallization dewetting) and dewetting due to the growth of perturbations in thin liquid films, originating from the intermolecular forces (spinodal dewetting) or impurities (heterogeneous nucleation). It was found that at high spin speeds, the film becomes ultrathin and dewets. The critical spin speed for dewetting depends on spin time and solution physical properties, as well.

The effect of the choice of solvent on PbCl_2_ film was studied, where it was found that a mixture of DMF and DMSO at a comparable volume ratio favors the formation of a film with high coverage. The XRD data showed that the perovskite film deposited using the one-step deposition shows the highest conversion of precursors to perovskite, whereas the film made using the two-step sequential spin-dip coating exhibited the highest absorbance. The device fabricated using the one-step method showed a PCE of 7.6 %, whereas the device made using the sequential spin-dip coating showed a PCE of 3.45 % but a higher fill factor. The SEM cross-sectional image of the one-step spin-coated cell showed excellent interfaces between the adjacent layers. However, during some steps of fabrication and testing, the devices were exposed to humid air (~50 %) and thus showed limited performance, showing the strong effect of humidity on perovskite solar cells.
